# The Cult of Middle-Age

**Published:** 1920-07-24

**Authors:** 


**THE PUBLIC WELL-BEING**—(*continued*).

### The Cult of Middle-Age.

### Trying to "Come Back."

The recent boxing match between Beckett, aged twenty-seven, and Burns, aged thirty-nine, seems to have attracted an interest far beyond the select coterie of those who practise "the noble art" and the wider circle of those who "follow" boxing. The explanation is that, in the public imagination, it was made a test case in the modern cult of middle age. In a sport so fierce and physically demanding as boxing—the acknowledged prerogative of youth—could a man verging on forty, however well preserved and carefully trained, justify his claim to stand up in equal fight to a champion in the prime of his young manhood? Even medical men took part in the discussion. One experienced member of the profession made a thorough examination of Burns at the climax of his training, and pronounced him as fit as a man could be at that age. He was sound as a bell, and his arteries were those of a man of twenty-eight or thirty! We know the result. In spite of these remarkable arteries Burns was easily beaten—actually in the seventh round, morally in the first.

The fact of the matter is that, all things being equal, it was a foregone conclusion. It has been the fashion in recent years for the middle-aged man to try to prove that he is as good as a man in the glorious twenties; but though this is a good thing in so far as it inspires a man to take care of himself and keep as young as possible, it is a bad thing when it tempts him to strain his heart and his nerve beyond reasonable limits. Almost without exception those who have tried to come back in athletics, even the "supermen," have failed. Jeffries failed, Driscoll failed. Our grey-headed and bald-headed lawn tennis champions, gallantly though they fight, lie in the dust before the onslaught of victorious youth. In Rugby football a man is usually an "old crock" at thirty, and who among Rugby men has not heard the finest players mournfully lament after that age how it "hurts" to be tackled forcefully and flung to the ground? Even in golf, essentially a game in which middle-aged men can prolong their activities successfully, the young, hard-hitting school start out with a long advantage over their older and more experienced but less supple and vigorous rivals. Running, leaping, and wrestling—they all belong to youth. Middle age is out of the running—the course of Nature is too deadly sure to give back this violent excess of energy after the appointed period is passed.

### Fallacious Theory.

The war has often been quoted in demonstration of the theory that a middle-aged man is as good as a young one. The older men, we were told, showed as much stamina, and certainly as much courage, as the younger ones. We read the other day of an old fellow of seventy who joined up in 1914 and fought in France up to the Armistice. It is true that middle age triumphantly asserted itself in the great campaigns in the West and showed an unexpected capacity to endure. It is true, indeed, that *unfit* middle-aged men performed prodigies of valour in the field. That very fact, however, seems to us to provide the real explanation. It must not be forgotten that the campaign of the Great War was totally different in one important respect from all others that preceded it. The health and fighting strength of the troops were maintained with a skill and care never before attempted. The medical service was a masterpiece of organisation. Sanitation was carried to a point of perfection. The system of reliefs was such that, though the soldiers had bad spells in the line, they had more or less regular spells in rest, when a reasonable amount of light training was combined with recreation of the best sort. They were well fed and well clothed. They had the heartening stimulus of periodical "leaves." Any man who reported sick was instantly examined and treated. The climate of Belgium and Northern France was akin to our own. All these factors tended to reduce the margin of effectivenesss between the young and middle-aged. The war clearly showed that the physique of the man of thirty-five or forty could be greatly developed and usefully maintained by systematic exercise, an open-air life, simple food, and hard manual work. It certainly proved nothing to justify his claim to compete with the ecstatic energy of youth itself.

These challenges which elderly aspirants after notoriety are so fond nowadays of flinging out through the public Press—coal-hewing contests between venerable members of Parliament, jaunty invitations from a famous author-sportsman of seventy-three to a former physician of sixty-nine to run a mile, swim a mile, scull a mile, box three rounds, and so forth—may serve to amuse; but they are valueless, and may easily be dangerous. The middle-aged are much more likely to live long and enjoy robust health if they give up morbid ambitions to beat youth in its own unchallengeable realm of sport and follow the sensible advice presented a few days ago by a "well-known dietetic expert" in the Press. Over-eating and the injudicious use of alcoholic drinks in middle age, he says, shorten the lives of a host of people. Unless a middle-aged man

THE PUBLIC WELL-BEING—(*continued*).

takes constant exercise he cannot digest and assimilate his heavy meals. The result is increased blood-pressure, self-poisoning through fermentation in the digestive organs, and the beginning of senile decay. We need not enlarge on the vital distinction between constant exercise and an attempt to knock out a British champion in the prize ring.

